# Wolfram syndrome 1b mutation suppresses Mauthner-cell axon regeneration via ER stress signal pathway

**DOI:** 10.1186/s40478-022-01484-8

**Published:** 2022-12-17

**Authors:** Zongyi Wang, Xinliang Wang, Lingyu Shi, Yuan Cai, Bing Hu

**Affiliations:** 1 Hefei National Research Center for Physical Sciences at the Microscale, Chinese Academy of Sciences Key Laboratory of Brain Function and Disease, Division of Life Sciences and Medicine, University of Science and Technology of China, Hefei, 230026 China; 2 Research Institute of Frontier Cross Science and Biomedical Sciences, Hefei Comprehensive National Science Center, Division of Life Sciences and Medicine, University of Science and Technology of China, Hefei, 230026 China; 3 First Affiliated Hospital of USTC, School of Life Sciences, Division of Life Sciences and Medicine, University of Science and Technology of China, Hefei, 230026 China

**Keywords:** wfs1b, Zebrafish, Mauthner cell, ER stress, Regeneration

## Abstract

Wolfram Syndrome (WS) is a fatal human inherited disease with symptoms of diabetes, vision decreasing, and neurodegeneration caused by mutations in the endoplasmic reticulum (ER)-resident protein WFS1. WFS1 has been reported to play an important role in glucose metabolism. However, the role of WFS1 in axonal regeneration in the central nervous system has so far remained elusive. Herein, we established a model of the *wfs1b* globally deficient zebrafish line. *wfs1b* deficiency severely impeded the Mauthner-cell (M-cell) axon regeneration, which was partly dependent on the ER stress response. The administration of ER stress inhibitor 4-Phenylbutyric acid (4-PBA) promoted M-cell axon regeneration in *wfs1b*^−/−^ zebrafish larvae, while the ER stress activator Tunicamycin (TM) inhibited M-cell axon regeneration in *wfs1b*^+*/*+^ zebrafish larvae. Moreover, complementation of *wfs1b* at the single-cell level stimulated M-cell axon regeneration in the *wfs1b*^*−/−*^ zebrafish larvae. Altogether, our results revealed that *wfs1b* promotes M-cell axon regeneration through the ER stress signal pathway and provide new evidence for a therapeutic target for WS and axon degeneration.

**Supplementary Information:**

The online version contains supplementary material available at 10.1186/s40478-022-01484-8.

## Introduction

Wolfram syndrome (WS) is an autosomal recessive neurodegenerative disorder, generally characterized by childhood-onset diabetes mellitus and optic nerve atrophy accompanied by hearing loss and diabetes insipidus [1–3]. WS patients had the clinical symptoms of visual field defects, cerebellar ataxia, epilepsy, anxiety, and cognitive impairment from neurological aspects [4, 5]. Subsequently, neuroimaging results showed that WS patients developed generalized brain atrophy, particularly in the cerebellum, medulla, and pons [6–11]. However, there is no effective treatment for WS.

The WFS1 protein, encoded by the *wfs1* gene, is an integral membrane protein located in the endoplasmic reticulum (ER), and the mutation of the *wfs1* gene is identified as the leading causative factor of WS. The functions of WFS1 have been extensively investigated in beta cells due to their abundance in the pancreas [6, 12–14]. This protein is also plentiful in the brain, particularly in the hippocampus, hypothalamus, cerebellum, and brainstem, indicating the prominent importance of WFS1 in the central nervous system (CNS) [15, 16]. Previous studies showed that the *wfs1*-deficient rats exhibited retinal gliosis, optic nerve atrophy, and medullary volume decrease, and retinal abnormalities were also found in the *wfs1*-deficient mice [17, 18]. At that time, a remarkable loss of retinal ganglion cells was detected in the *wfs1b* mutant zebrafish retinas [19]. In addition, knockdown of *wfs1* in neurons in the fly brain led to age-dependent behavioral deficits and neurodegeneration [20]. More interestingly, WFS1 might participate in the pathogenesis of Alzheimer’s disease (AD) [21–24]. However, relatively little is known about the link between WFS1 and axon regeneration.

WFS1 is also a component of the unfolded protein response (UPR), which is a series of signal transduction pathways caused by ER stress response [25]. The mRNA expression level of *wfs1* was upregulated in response to ER stress [26]. Concurrently, the ER stress response was stimulated by the WFS1 deficiency, which led to dysfunction of the pancreatic islets and the nervous system in the *wfs1*-deficient rats and WS patients, indicating that WFS1 is related to the ER stress response [6, 11–14, 17, 27].

The M-cell is a single pair of motor neurons in the brain stem of zebrafish which regulates the escape response triggered by abrupt stimuli [28, 29]. According to recent studies, M-cells show a strong ability to regenerate in zebrafish in contrast to the inability to regenerate in the adult mammalian CNS [30–34], allowing zebrafish to become an emerging model to investigate spinal-cord injury (SCI), which remains a major cause of morbidity and societal expense on account of its global prevalence [35, 36]. SCI and degenerative CNS disorders may cause axon degeneration, which is regarded as a therapeutic target for treating neurodegenerative diseases [37, 38]. Therefore, we intend to investigate M-cell axon regeneration using the SCI model in zebrafish, allowing us to better understand axonal degeneration in the degenerative CNS disease of WS and provide potential therapy for WS patients. Additionally, zebrafish is an ideal model for the study of human diseases because zebrafish have at least one obvious orthologue for over 70% of human genes, which means zebrafish are genetically similar to humans [39].

Here, we established a mutant zebrafish line that has a 23-bp deletion in *wfs1b* and showed that loss of *wfs1b* inhibited M-cell axon regeneration, which is partly caused by *wfs1b* deficiency-mediated ER stress response. Together, our study showed that *wfs1b* is essential for ER stress response that regulates M-cell axon regeneration, providing a new therapeutic target for WS.

## Materials and methods

### Zebrafish strains and maintenance

Adult zebrafish were maintained in an aquatic habitat system at 28.5 °C with a light/dark cycle of 14/10-hour (14-h light and 10-h dark cycle). Embryos were collected after natural spawning and raised at 28.5 °C in an incubator (5 mM NaCl, 0.17 mM KCl, 0.33 mM CaCl_2_, 0.33 mM MgSO_4_, and 0.1% methylene blue, pH 7.0). From 24 hpf, embryos were supplemented with 0.003% N-phenylthiourea (PTU, Sigma-Aldrich, USA) to avoid pigmentation. The transgenic line employed in this study is Tg (Tol 056: EGFP), in which M-cells express Enhanced Green Fluorescence Protein (from RIKEN, Japan). The University of Science and Technology of China (USTC) Animal Resources Center and the University Animal Care and Use Committee provided the rules and regulations that all experiments implemented. All protocols were subject to approval by the Committee on the Ethics of Animal Experiments of the USTC (permit no. USTCACUC1103013).

### Genome editing

CRISPR-mediated genome editing for the generation of *wfs1b*^*−/*−^ mutants was performed. Cas9 mRNA was synthesized accordingly using appropriate plasmids (108301; Addgene) with the mMessage mMachine T7 Ultra Kit (Thermo Fisher). The single-guide RNA (sgRNA) targeting the *wfs1b* sequence was synthesized using the plasmids mentioned above with the Megashortscript T7 kit (Thermo Fisher). We blended the Cas9 mRNA (300 ng/μL) with the sgRNA (40 ng/ μL) gently and then microinjected this mixture into one-cell stage embryos.

### Single-cell electroporation

Before electroporation, 4 dpf larvae were anesthetized with ethyl 3-aminobenzoic methanesulfonate (MS222, Sigma-Aldrich) and embedded in 1% low-melting agarose (Sangon, China) in an electroporation chamber. A micropipette tip pulled by a micropipette puller (Sutter Instrument, USA) was filled with plasmids and placed near the M-cell soma. Electric stimulation was applied to the zebrafish larvae, delivering the plasmids, whose concentration is 120 ng/μL, into the unilateral M-cell.

### Two-photo axotomy

Anesthetic zebrafish larvae at 6 dpf were fixed in 1% low-melting agarose in a chamber prior to axotomy. A Zeiss microscope (LSM710; Carl Zeiss, Oberkochen, Germany) equipped with a two-photon was used at a wavelength of 800 nm and an intensity of 15%–35% to ablate the M-cell axons under a 25 × oil dipping lens.

### In vivo imaging

Larvae were sedated with MS222 and then embedded in 1% low-melting agarose in a chamber. Larvae were photographed at 2 days after axotomy with a confocal system (FV1000; Olympus, Tokyo, Japan) and a water dipping lens (40 × , 0.85 numerical-aperture objective). Z-stack images were acquired at 3-μm intervals.

### Quantitative real-time PCR

Total RNA was extracted from the whole larvae using RNAsio (TAKARA), and approximately 1 μg of RNA was reverse-transcribed into cDNA using HiScript II qRT SuperMix II (Vazyme). qPCR application was performed in a total volume of 10 μL containing 5 μL ChamQ Universal SYBR Green qPCR Master Mix and 1 μL cDNA template on a real-time quantification system (LightCycler 96, Roche). The mRNA expression levels were analyzed using the comparative Ct relative quantification method formula 2^−△△CT^, with housekeeping gene β-actin mRNA used as invariant control to normalize the mRNA of target genes, which was repeated three times for each sample. All primers used are listed in Additional file 1: Supplementary Table S1.

### Protein extraction and western blotting

Wildtype and *wfs1b*^*−/−*^ mutant larvae at 5 dpf were collected and lysed with RIPA buffer supplemented with protease inhibitor and phosphatase inhibitor (Sangon). The lysates were centrifuged and the collected supernatant was kept on ice. Using BCA Protein Assay Kit (Beyotime) in accordance with the manufacturers’ instructions, the concentration of each protein sample was assessed on a microplate reader.

Samples were boiled for 4 min and run on a 10% SDS-PAGE gel with loading buffer (5 ×) and transferred to PVDF membrane. After incubation in 5% nonfat milk and TBST for 60 min at room temperature, the membranes were washed once with TBST and incubated with antibodies against WFS1 (1:1000; Proteintech) or β-Actin (1:2000; HuaAn) at 4 °C for 12 h. Subsequently, the membranes were incubated with secondary goat anti-rabbit antibodies (1:5000; Proteintech) for 1 h at room temperature. Blots were washed with TBST three times and visualized by enhanced chemiluminescence (ECL) system (Thermo Fisher). The densities of bands were quantified by ImageJ software and normalized to protein β-Actin.

### Whole-mount in situ hybridization

Whole-mount in situ hybridizations were performed as described [40]. A partial fragment of *wfs1b* was amplified from cDNA generated from 5 dpf RNA with primers listed in Table S1 for the sake of preparing for the synthesis of digoxygenin (DIG)—labeled RNA probes. DIG-labeled RNA probes were synthesized using DIG RNA labeling mix (Roche) and T7 RNA polymerase (Thermo Fisher). A series of in situ hybridization was replicated three times with the use of independently collected embryos.

### Drug treatment

The 4-Phenylbutyric acid (4-PBA) (P21005, Sigma-Aldrich) is a well-characterized ER stress antagonist while Tunicamycin (TM) (654,380, Sigma-Aldrich) is a well-known ER stress activator [41, 42]. We pretreated larvae with 4-PBA at a final concentration of 50 μM from 24 hpf to 8 dpf [2 days post axotomy(dpa)] and pretreated larvae with TM at a final concentration of 0.5 μg/ml from 4 to 8 dpf during all of the regeneration experiments. The treatment of EM containing DMSO and PTU was applied in the control larvae.

### Transmission electron microscopy

Brain tissues were taken from 6 dpf zebrafish and immediately fixed in 2.5% glutaraldehyde solution overnight at 4 °C. After washing with PBS, specimens were then incubated in the post-fixation solution containing 1% osmic acid for 2 h. Subsequently, specimens were dehydrated with serial dilutions of ethanol in water (50%, 70%, 80%, 95%) for 10 min and 100% ethanol twice for 50 min each. The samples were then embedded in Epon resin with surrounding support tissue and polymerized at 45 °C for 12 h and at 72 °C for 24 h. Ultrathin (70 nm) transverse sections of the brain were segmented by Leica UC-7 and stained with uranyl acetate and lead citrate. Sections were viewed and photographed with a JEM-1400 transmission electron microscope (TEM). Statistical methods were utilized by reference to the previous study [43].

### Optokinetic response assay

The optokinetic response (OKR) behavior test was accomplished according to the previous study in order to examine the visual function [44, 45]. A sine-wave grating, which is projected by an LCD projector (NEC 280+; NEC Corporation, Japan), was generated by the software LabVIEW. 5 dpf Zebrafish larvae were placed dorsal side up in 6% methylcellulose solution to hinder from body movement. An infrared-sensitive CCD camera (TCA-1.3BW; Nanjing, China) monitored the elicited eye movement in real time while the rotating grating was placed around the larvae. Wildtype and *wfs1b* mutant larvae were stimulated with a constant angular velocity of 7.5 degree/s and a fixed spatial frequency (SF) of 0.06 cycles/degree. The gain which equals the ratio of eye velocity and stimulus velocity was used to gauge contrast sensitivity.

### Escape behavior assay

The device system is composed of a high-speed camera (1000 fps), a computer, a loudspeaker. The 8 dpf zebrafish larvae were placed in a petri dish with EM and moved to a platform with appropriate light around. The computer was connected to a loudspeaker near the petri dish and a high-speed camera were adjusted properly. Before each test, the larvae were left for 5 min without being disturbed. Movement trajectory was induced by sound stimulation of sinusoidal waves (500 Hz, 20 ms) and video acquisition was controlled by specialized software. For injured group, unilateral M-cells were ablated with two-photon axotomy in 6 dpf before escape behavior assay.

### Statistical analysis

Graphs and statistical significance were analyzed with GraphPad Prism 8.0 software (San Diego, USA), Adobe Photoshop CC2020, and Adobe Illustrator CC 2020. Data are presented as the mean ± standard error of the mean (SEM). Experiments were analyzed using unpaired two-tailed Student’s *t*-tests. Experiments with more than two groups were analyzed using one-way analyses of variance (ANOVAs), and experiments involved two independent variables using two-way ANOVAs. Experiments were repeated at least three times. Differences were considered significant when *P ≤ 0.05, **P ≤ 0.01, and ***P ≤ 0.001, ****P ≤ 0.0001. The figure legends provided all other pertinent information, such as sample size and precise statistical tests used.

## Results

### Developmental Expression and Characteristics of *wfs1b* in zebrafish larvae.

**Fig. 1:**
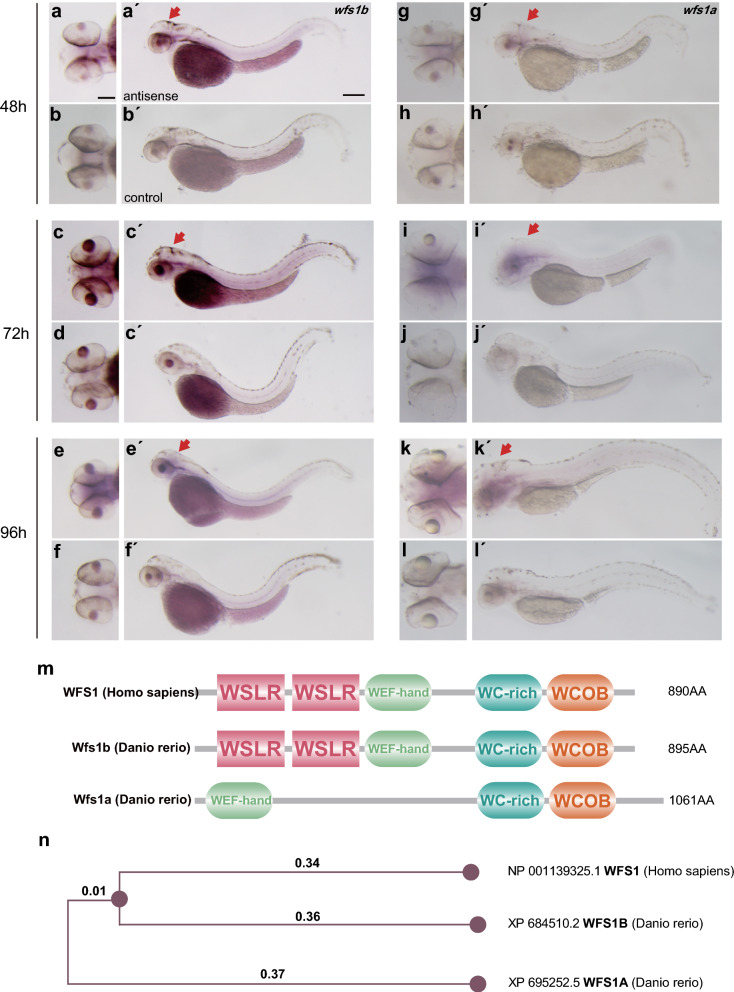
*wfs1a* and* wfs1b* genes in zebrafish larvae. **a**–**f** Expression pattern of *wfs1b* as shown by whole-mount in situ hybridization. **a′**–**f′** were the images of *wfs1b* expression in the brain of zebrafish larvae. **g**–**l** Expression pattern of *wfs1a* as shown by whole-mount in situ hybridization. **g**′–**l**′ were the images of *wfs1a* expression in the brain of zebrafish larvae. The dorsal view (scale bar, 5 μm) and lateral view (scale bar, 10 μm) of the zebrafish larvae at different stages showed that *wfs1b* is expressed in the CNS (red arrows) as well as wfs1a. **m** The predicted protein structure of WFS1 between human and zebrafish. **n** The phylogenetic tree with the maximum-likelihood algorithm. The numbers represent the length of the evolutionary branch and it determines the degree of homology, Wfs1b has a shorter distance compared to Wfs1a, which means Wfs1b has higher evolutional conservation

The *wfs1* gene, which is only present as a single copy in humans, is present in two copies in zebrafish. Using whole-mount in situ hybridization (WISH), we assessed the pattern of mRNA expression of *wfs1a* and *wfs1b* at various development stages. Two *wfs1* gene transcripts were examined at 48, 72, and 96 hpf, and during the developmental stage, they were all expressed throughout the CNS (Fig. 1a-l and a′-l′). Similar mRNA expression patterns were reported in their zebrafish embryos [46]. The Wfs1b protein consisted of 895 amino acids while the Wfs1a protein consisted of 1061 amino acids. A computational analysis by the Pfam database (pfam.xfam.org) predicted the domains of protein among Wfs1b, Wfs1a, and WFS1. Wfs1b has similar domains to WFS1 (Fig. 1m). The alignment of correlative amino acid sequences resulted in the creation of a phylogenetic tree with neighbor-joining and maximum-likelihood algorithms through MEGA-11 software. There was higher evolutionary conservation between the WFS1 in humans and the Wfs1b in zebrafish (Fig. 1n).

### Identification of *wfs1b* mutant zebrafish

**Fig. 2:**
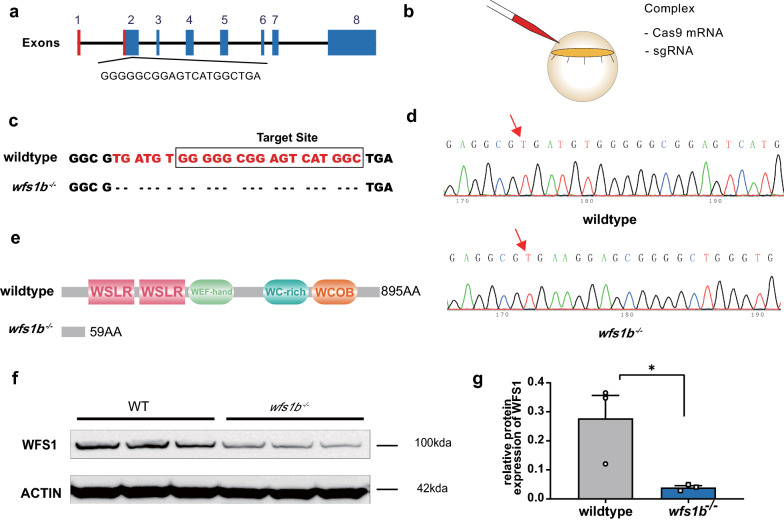
Generation and identification of *wfs1b* mutant zebrafish. **a** Schematic of the Cas9-sgRNA targeted site located at the second exon of *wfs1b*. **b** schematic of the complex injected into one-cell embryos. **c**, **d** Representative sequencing results of wide-type and mutated zebrafish lines. The mutant sequencing result showed a 23-bp base deletion. **e** Bioinformatics analysis indicated that the mutated region is located in the front of the functional domain of Wfs1b. The wildtype translated to 895 amino acids, whereas the mutant translated to 59 normal amino acids. **f**, **g** Western-blotting analysis showed that WFS1 protein expression is inhibited in the mutant group compared with that of the wildtype. The protein expressions were quantified by Image J software. The experiment was repeated three times with three independent samples. *P* = 0.0394. Assessed by unpaired *t* test

Since zebrafish *wfs1b* has higher conservation, we then focused on *wfs1b* and designed a CRISPR-Cas9-targeted site in its second exon (Fig. 2a). The *wfs1b* sgRNA and Cas9 mRNA were transcribed and then co-microinjected into one-cell embryos (Fig. 2b). After two generations of gene identification and larvae cultivation, we obtained a homozygous *wfs1b* mutant zebrafish line. To precisely evaluate the type of mutation, we amplified about 678-base pair (bp) DNA fragment from genomic DNA by polymerase chain reaction (PCR) and then digested the DNA fragment with EciI endonuclease. Gel electrophoresis findings revealed that F2 zebrafish larvae were heritably homozygous mutants (Additional file 2: Supplementary Fig. S1). According to the sequencing results, a 23-bp loss was detected, which led to a premature termination codon in its third exon (Fig. 2c, d). In addition, we amplified genomic DNA and complementary DNA (cDNA) fragments with primers spanning the target site, and the results showed that no PCR band was displayed in *wfs1b*^*−/−*^ groups (Additional file 2: Supplementary Fig. S2). The functional sequence domains of the F2 zebrafish Wfs1b protein were also found to be frame-shifted, according to bioinformatics analysis (Fig. 2e). Lastly, Western blot data revealed that the expression of WFS1 protein was decreased in *wfs1b*^*−/−*^ mutant zebrafish (Fig. 2f, g). Meanwhile, the OKR behavior test was utilized to assess the visual function, and the test indicated that the visual function was obviously damaged in 5 dpf *wfs1b*^−/−^ zebrafish (Additional file 2: Supplementary Fig. S3). Thus, the results above verified that we successfully generated a *wfs1b*^*−/−*^ mutant zebrafish line.

### Deficiency in Wfs1b suppresses Mauthner-cell axon regeneration in vivo

**Fig. 3:**
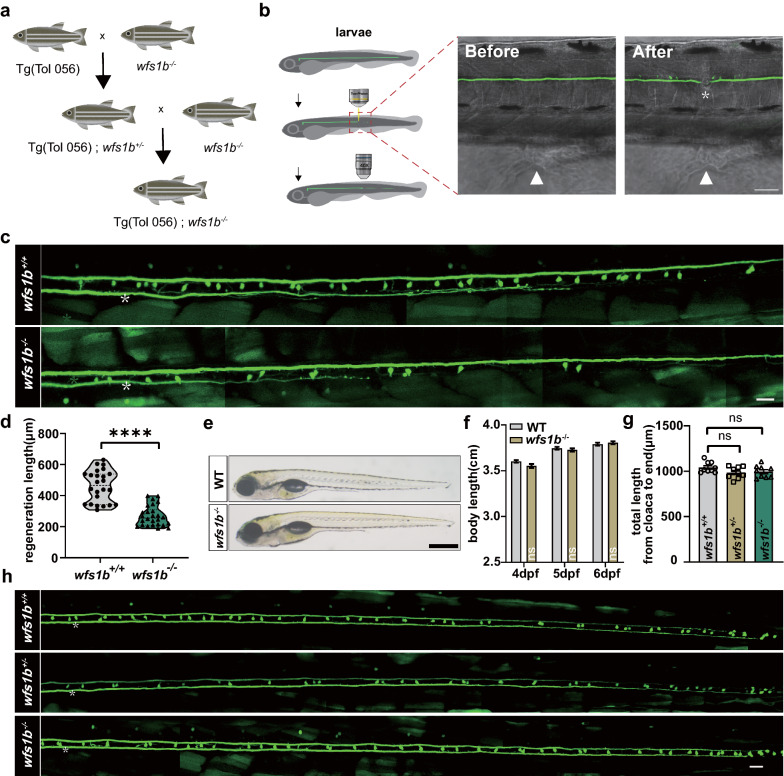
Wfs1b regulates M-cell axon regeneration in vivo. **a** Hybridization of the transgenic line: Tg (Tol 056: EGFP) and *wfs1b* mutants were crossed for two consecutive generations to obtain Tg (Tol 056: EGFP)/ *wfs1b* ^+/-^ and Tg (T056: EGFP); *wfs1b* ^−/−^ lines. **b** Representative images of the M-cell axon before and after ablations by a two-photon laser. Asterisk, injury site; arrowhead, cloacal pore; scale bar, 50 μm. **c**, **d** Confocal imaging of M-cell axons between* wfs1b*^*+/+*^ and *wfs1b*^*−/−*^ groups at 8 dpf and the regeneration length at 2 dpa. Violin plot shows all data points, including minimum, maximum, median, and quartiles. Scale bar, 20 μm, P < 0.0001, control, *n* = 24; *wfs1b*^*−/−*^, *n* = 26. Assessed by unpaired *t* test. **e**, **f** Representative images of embryos from the wildtype and the mutant at 6 dpf (scale bar, 500 μm), and measured total body length from 4 to 6 dpf before axotomy (4 dpf, wildtype: 3.603 ± 0.01402 cm, *wfs1*^*−/−*^: 3.549 ± 0.02201 cm, *P* = 0.1708; 5 dpf, wildtype: 3.745 ± 0.01504 cm, *wfs1*^*−/−*^: 3.727 ± 0.01679 cm, *P* = 0.8255; 6 dpf, wildtype: 3.790 ± 0.01691 cm, *wfs1*^*−/−*^: 3.806 ± 0.01569 cm, *P* = 0.8842; *n* = 30). Assessed by two-way ANOVA. ns, not significant. **g**, **h** Defined lengths of M-cell axons from the cloaca to the end were not notably different among WT, homozygous, and heterozygous larvae (*wfs1b*^+*/*+^: 1042 ± 19.51 μm; *wfs1b*^+/-^: 985.2 ± 21.47 μm, *P* = 0.0689; *wfs1b*^*−/−*^: 995.6 ± 21.98 μm, *P* = 0.1363; *n* = 9). Assessed by ordinary one-way ANOVA/Tukey’s multiple-comparisons test (*wfs1b*^+*/*+^ versus *wfs1b*^+/-^: *P* = 0.1594; *wfs1b*^+*/*+^ versus *wfs1b*^*−/−*^: *P* = 0.2856; *wfs1b*^+/-^ versus *wfs1b*^*−/−*^: *P* = 0.9342) White asterisk: ablation point. Scale bar, 20 μm. ns, not significant

Previous studies have shown the M-cells’ ability for regeneration [32–34]. The transgenic line Tg (Tol 056: EGFP) was mated with the *wfs1b* mutant line and the Tg (Tol 056: EGFP)/ *wfs1b*^*−/−*^ zebrafish line was obtained so as to explore what role *wfs1b* plays in M-cell axon regeneration (Fig. 3a). One of the M-cell axons was transected over the cloacal pores at 6 dpf utilizing two-photon laser axotomy (Fig. 3b). When compared to wildtype (*wfs1b*^+*/*+^) larvae, in vivo live imaging at 8 dpf revealed that *wfs1b*^*−/−*^ mutant larvae exhibited a reduction in the length of M-cell axon regeneration, indicating that the regenerative capacity of the *wfs1b*^*−/−*^ mutant is inferior to the wildtype (control: 461.7 ± 20.82 μm; *wfs1b*^*−/−*^: 268.8 ± 12.84 μm; Fig. 3c, d). We calculated the whole-body length from 4 to 6 dpf and the length of M-cell axons from the cloaca to the end and observed no discernible differences among wildtype, *wfs1b*^+/−^, and *wfs1b*^*−/−*^ mutant larvae. This demonstrated that the *wfs1b* mutant had no impact on the development of the M-cell axon itself (Fig. 3e–h). Previous research has proved that heterozygous carriers of the gene for the WS are vulnerable to psychiatric illness and *wfs1*^+/−^ mice exhibited higher sensitivity to the high-fat diet [47, 48], we assumed that the ability of M-cell axon regeneration in the *wfs1b*^+/−^ zebrafish might be affected. Then we found that *wfs1b*^+/−^ mutant larvae displayed a reduction in the length of M-cell axon regeneration as well (control: 472.5 ± 20.65 μm; *wfs1*^+/-^: 356.2 ± 21.59 μm; Additional file 2: Supplementary Fig. S4).

**Fig. 4:**
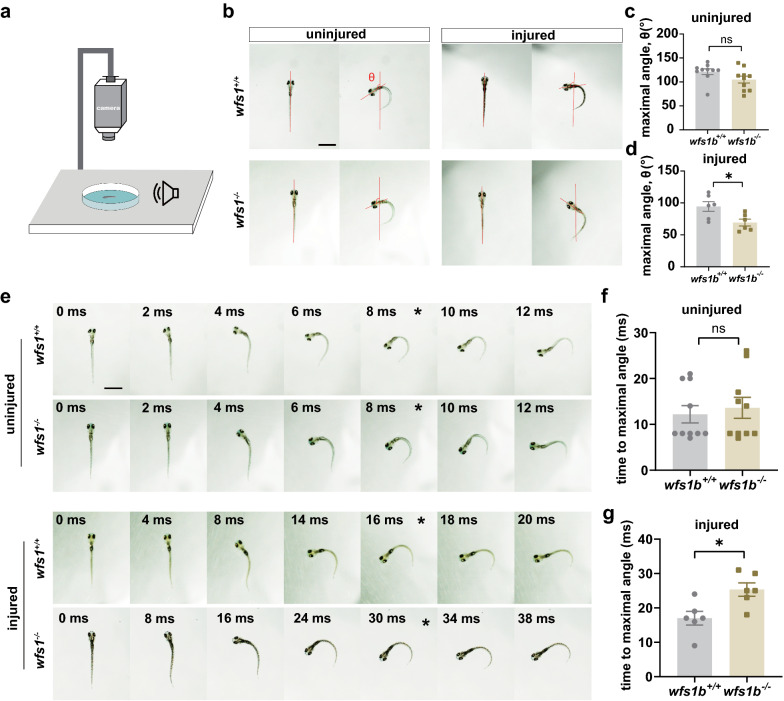
Escape Behavior Test Exhibited the Impaired Function of M-cell. **a** Device for testing escape behavior. **b** Representative images of the original orientation and maximal turn angle position from the *wfs1b*^+*/*+^ and *wfs1b*^*−/−*^zebrafish larvae in the uninjured and injured groups. Red lines indicate the heading direction. **c**, **d** Statistical diagram of maximal turn angle, θ. uninjured: P = 0.0921 (*wfs1b*^+*/*+^:121.5° ± 5.879; *wfs1b*^*−/−*^:104.9° ± 7.200); injured: P = 0.0230(*wfs1b*^+*/*+^:94.44° ± 7.681; *wfs1b*^*−/−*^:69.31° ± 5.465); n = 10; ns, not significant. Scale bar, 1 mm. Assessed by unpaired t test. **e** A series of images of movement trajectory from the *wfs1b*^+*/*+^ and *wfs1b*^*−/−*^zebrafish larvae in the uninjured and injured groups. asterisk: maximal turn angle position. **f**, **g** Statistical diagram of time to maximal turn angle. uninjured, P = 0.6410 (*wfs1b*^+*/*+^:12.2 ± 1.879 ms; *wfs1b*^*−/−*^:13.6 ± 2.276 ms); injured, P = 0.0133 (*wfs1b*^+*/*+^:17.00 ± 1.983 ms; *wfs1b*^*−/−*^:25.33 ± 1.944 ms); n = 6; ns, not significant. Scale bar, 1 mm. Assessed by unpaired t test

M-cells are relevant to escape behavior, which is considered to have two obvious stages: the C-start and escape swimming [28]. In order to investigate the function of M-cells in the *wfs1b*^*−/−*^ zebrafish larvae, an experimental device system was set up to monitor the movement trajectory (Fig. 4a). Two indicators were measured on the basis of the referenced methods: maximal turn angle and time to the maximal turn angle [49, 50]. Our results showed that the maximal turn angle was shorter when a unilateral M-cell was transected after 48 h and the time to the maximal turn angle was longer in the *wfs1b*^*−/−*^ zebrafish larvae, whereas there was no obvious difference when M-cells were not transected between the control and *wfs1b*^*−/−*^ groups (Fig. 4b–g).

### Complementation of *wfs1b* promotes axon regeneration at a single cell level

**Fig. 5:**
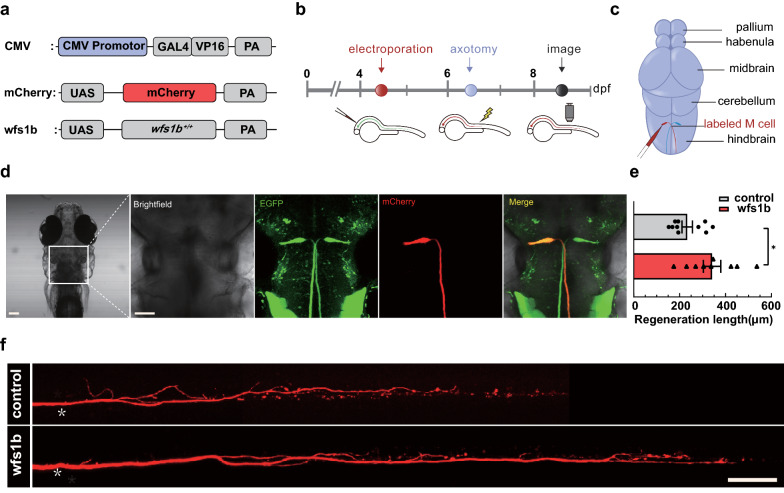
Complementation of *wfs1b* at single-cell electroporation promotes M-cell axon regeneration. **a** Construction of the *wfs1b* expression system. Plasmids express only mCherry served as the control vector. **b** Timeline of time points of electroporation, axotomy, and regeneration imaging. **c** Schematic of M-cell soma electroporation. **d** Confocal imaging of zebrafish larvae 12 h after electroporation (far left) and magnified images of the brain stem in zebrafish larvae, denoting the position of M-cell soma under different fluorescence in the white box. mCherry represents the axons that were labeled by mCherry. Scale bar, 50 μm. **e**, **f** *wfs1b* gene retro-complementation rescued the length of Mauthner cell axon regeneration (control: 233.1 ± 22.65 μm, *n* = 10; *wfs1b* overexpression: 341.4 ± 37.92 μm, *n* = 9). White asterisk: ablation point. scale bar, 50 μm. *P* = 0.0225. Assessed by unpaired *t* test

To explore more about the function of *wfs1b* in M-cell axon regeneration further, we carried out cell-type-specific retro-complementation. Plasmids containing UAS-*wfs1b* and UAS-mCherry were constructed (Fig. 5a). Thereafter, plasmids CMV-GAL4-VP16/UAS-mCherry (served as control group) and CMV-GAL4-VP16/UAS-mCherry/ UAS-*wfs1b* were co-transfected into unilateral M-cell through single-cell electroporation at 4 dpf in *wfs1b*^*−/−*^ mutant zebrafish respectively (Fig. 5b, c). Zebrafish larvae, whose M-cell exhibited red-fluorescent that mCherry fluorescent protein expressed, were selected 12 h after electroporation (Fig. 5d). Subsequently, we ablated red-fluorescent M-cell axons at 6 dpf with a two-photon laser scanning microscope and proceeded to image the regenerated length of M-cell axons at 8 dpf (2 dpa). After complementation of the *wfs1b* gene in a single M-cell axon, the capacity for regenerate was activated (Fig. 5e, f).

### ER stress response was evoked due to loss of Wfs1b

**Fig. 6:**
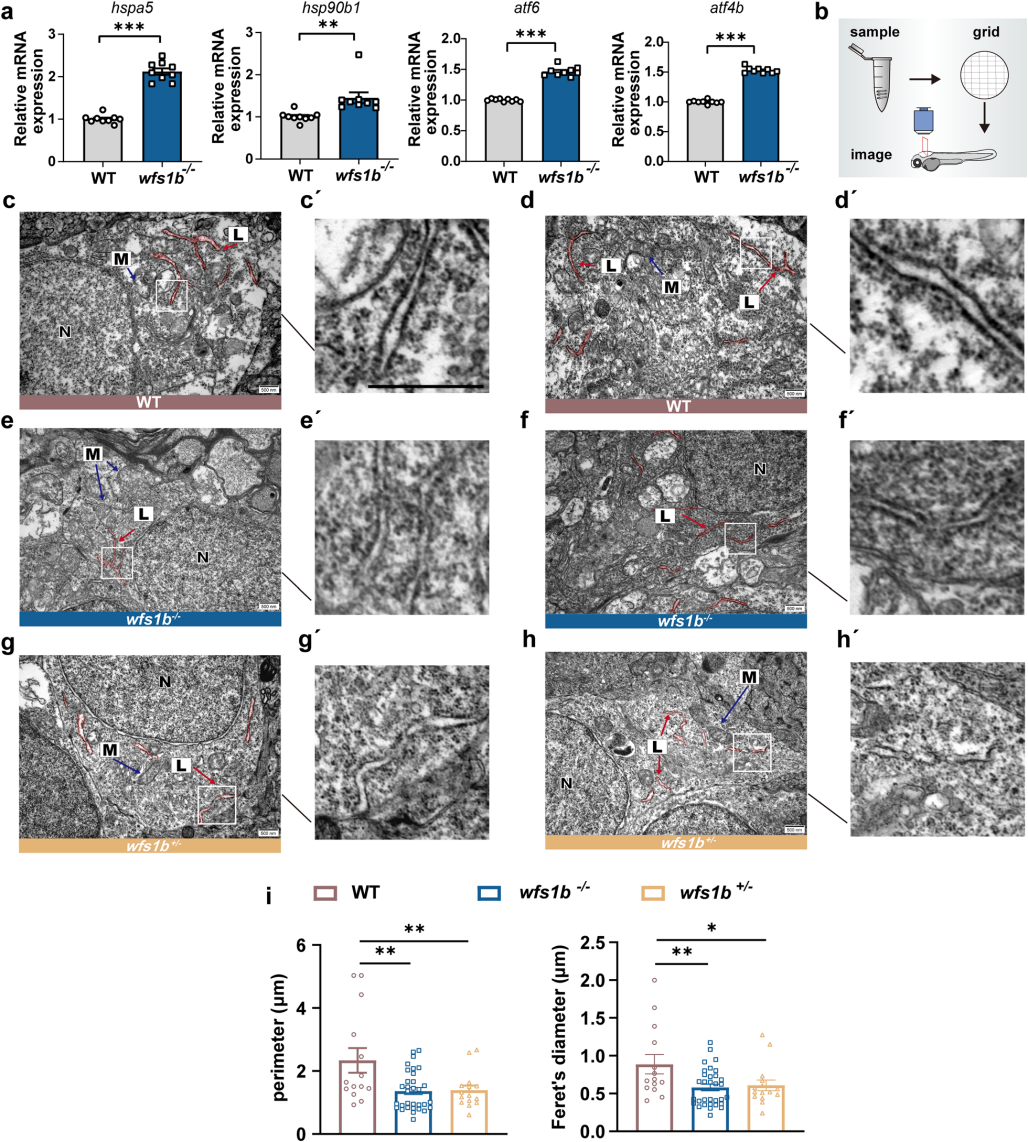
Loss of Wfs1b provokes the ER stress response. **a** qPCR analyses of up-regulated downstream genes of the UPR pathway. *hspa5*, *P* < 0.0001; *hsp90b1*,* P* = 0.0046; *atf6*, *P* < 0.0001; *atf4b*, *P* < 0.0001. Assessed by unpaired *t* test. **b** Diagram of the electron microscope’s operation. **c**–**h** Representative TEM imaging showed the ER morphology in wildtype (upper), *wfs1b*^*−/−*^ mutant (middle) and *wfs1b*^±^ mutant (bottom) larvae brain, indicating that the ER morphology was ruptured. (C′-H′) were the ER ultrastructure under magnification from the white boxes in **c**–**f**. Scale bar, 500 nm. L, rough endoplasmic reticulum; N, cell nuclei; M, mitochondria. **i** Statistical diagram of perimeter and Feret’ diameter among the wildtype, the *wfs1b*^*−/−*^ and *wfs1b*^+/-^ mutant zebrafish larvae. Assessed by ordinary one-way ANOVA

Considering Wfs1b is mainly localized in the ER, we hypothesized that *wfs1b* knockout might affect the ER structure and stimulate the ER stress-associated signaling pathway. The UPR is comprised of three pathways: IRE1 (inositol requiring enzyme 1), PERK [double-stranded RNA-activated protein kinase (PKR)–like ER kinase], and ATF6 (activating transcription factor 6) [51, 52]. The expression levels of the UPR pathway were detected by qPCR and we found that the transcriptional level of several UPR-relevant genes, including *hspa5*, *hsp90b1*, *atf6*, and *atf4b*, were upregulated in *wfs1b* mutant zebrafish compared to the control group (Fig. 6a). Meanwhile, ER morphology in the zebrafish brain was observed employing Electron Microscopy (Fig. 6b). TEM imaging results showed that the ER structure was fractured both in homozygous and heterozygous *wfs1b* zebrafish (Fig. 6c–i). Abnormality of ER morphology was also observed in *wfs1*^*−/−*^ rodent models and patients [14, 53, 54]. Above all, our findings indicated that *wfs1b* and ER stress are related.

### *wfs1b* deficiency-induced ER stress affects Mauthener-cell axon regeneration

**Fig. 7:**
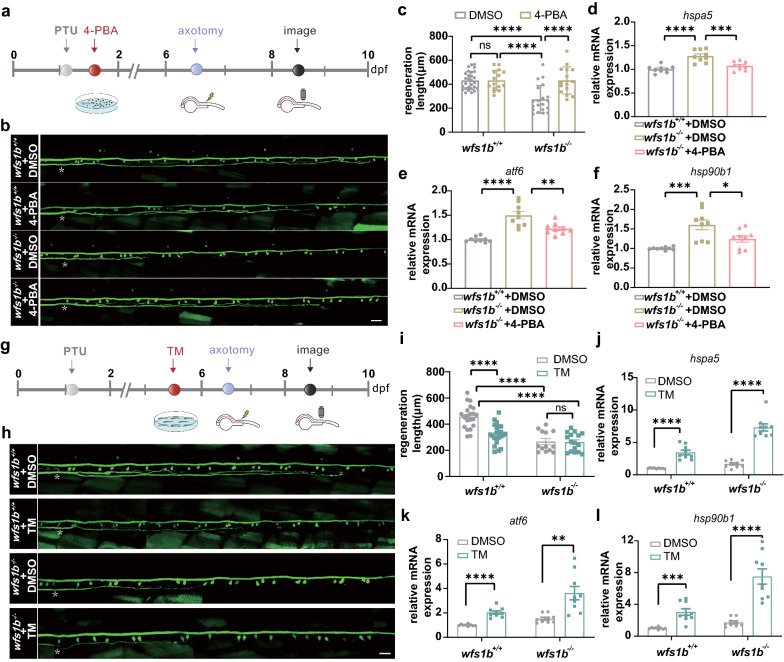
ER Stress Response Affects M-Cell Axon Regeneration. **a** Timeline of time points of 4-PBA treatment, axotomy, and regeneration imaging. **b**, **c** Representative confocal imaging of Mauthner-cell axon in *wfs1b*^+/+^ treated with DMSO, *wfs1b*^+/+^ treated with 4-PBA, *wfs1b*^*−/−*^ treated with DMSO, and *wfs1b*^*−/−*^ treated with 4-PBA at 8 dpf and the regeneration length at 2 dpa (*wfs1b*^+*/*+^  + DMSO: 431.7 ± 14.12 μm, *n* = 28; *wfs1b*^+*/*+^  + 4-PBA:432.7 ± 21.63 μm, *n* = 15; *wfs1b*^*−/−*^ + DMSO: 274.2 ± 25.80 μm, *n* = 20; *wfs1b*^*−/−*^ + 4-PBA: 432.0 ± 29.26 μm, *n* = 15). Assessed by two-way ANOVA/Tukey’s multiple-comparisons test (*wfs1b*^+*/*+^  + DMSO versus *wfs1b*^+*/*+^  + 4-PBA:* P* > 0.9999; *wfs1b*^+*/*+^  + DMSO versus *wfs1b*^*−/−*^ + DMSO: *P* < 0.0001; *wfs1b*^*−/−*^ + DMSO versus *wfs1b*^*−/−*^ + 4-PBA: *P* < 0.0001; *wfs1b*^+*/*+^  + 4-PBA versus *wfs1b*^*−/−*^ + DMSO: *P* < 0.0001). Scale bar, 20 μm. ns, not significant. **d**–**f** qPCR analyses of downstream genes of the UPR pathway. Assessed by ordinary one-way ANOVA. **g** Timeline of time points of TM treatment, axotomy, and regeneration imaging. **h**, **i** Representative confocal imaging of Mauthner-cell axon in *wfs1b*^+/+^ treated with DMSO, *wfs1b*^+/+^ treated with TM, *wfs1b*^*−/−*^ treated with DMSO, and *wfs1b*^*−/−*^ treated with TM at 8 dpf and the regeneration length at 2 dpa (*wfs1b*^+*/*+^  + DMSO: 456.9 ± 19.27 μm, *n* = 21; *wfs1b*^+*/*+^  + TM: 323.4 ± 15.80 μm, *n* = 23; *wfs1b*^*−/−*^ + DMSO: 266.1 ± 21.94 μm, *n* = 13; *wfs1b*^*−/−*^ + TM: 263.2 ± 17.29 μm, *n* = 16). Assessed by two-way ANOVA/Tukey’s multiple-comparisons test (*wfs1b*^+*/*+^  + DMSO versus *wfs1b*^+/+^  + TM: *P* < 0.0001; *wfs1b*^+*/*+^  + DMSO versus *wfs1b*^*−/−*^ + DMSO: *P* < 0.0001; *wfs1b*^+*/*+^  + DMSO versus *wfs1b*^*−/−*^ + TM:* P* < 0.0001; *wfs1b*^*−/−*^ + DMSO versus *wfs1b*^*−/−*^ + TM: *P* = 0.9997). Scale bar, 20 μm. ns, not significant. **j**–**l** qPCR analyses of downstream genes of the UPR pathway. Assessed by unpaired *t* test. White asterisk: ablation point

To better understand whether ER stress is involved in M-cell regeneration, we tested the involvement of ER stress in this model via a pharmacological approach. ER stress inhibitor 4-PBA was applied at 1 dpf (Fig. 7a), and *wfs1b* mutant zebrafish larvae pretreated with 4-PBA had boosted M-Cell axon regeneration while there was no obvious difference in *wfs1b*^+*/*+^ zebrafish (Fig. 7b, c). In addition, decreased mRNA expression of relevant ER stress genes at 6 dpf was observed, while the expression of *atf4b* did not decrease after treatment with 4-PBA (Fig. 7d–f and Additional file 2: Supplementary Fig. S5A). It should be noted that the application of 4-PBA didn’t relieve the abnormal morphology of ER (Additional file 2: Supplementary Fig. S6). The *wfs1b*^+*/*+^ zebrafish group served as a blank control. Then, the *wfs1b*^+*/*+^ zebrafish larvae pretreated with ER stress inducer TM had diminished M-Cell regeneration (Fig. 6g–i) while there was no obvious difference between *wfs1b* mutant zebrafish groups. qPCR analyses confirmed that the application of TM evoked ER stress response both in the *wfs1b*^+*/*+^ and *wfs1b*^*−/−*^ zebrafish larvae (Fig. 7j–l and Additional file 2: Supplementary Fig. S5B). Together, these data demonstrated that ER stress is associated with M-Cell axon regeneration.

## Discussion

**Fig. 8:**
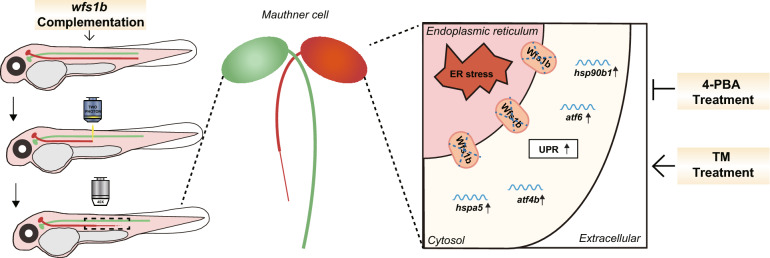
Overview of *wfs1b* Deficiency-Mediated ER Stress Inhibits M-cell Axon Regeneration. The *wfs1b* knockout impeded the M-cell axon regeneration due to increased ER stress, resulting in up-regulated mRNA expression of the UPR pathway. The ER stress activator TM inhibited M-cell axon regeneration in *wfs1b*^+*/*+^ zebrafish larvae, whereas both the application of ER stress inhibitor 4-PBA and complementation of *wfs1b* at the single-cell level ameliorated the degenerated M-cell axon in *wfs1b*^*−/−*^ zebrafish larvae

WS is considered as a rare neurodegenerative disorder associated with ER dysfunction and its main causative gene *wfs1* has been widely reported on its function in pancreatic beta cells but barely in the CNS [1, 12–14]. Here, we focused on the impact of *wfs1b* on axonal regeneration. As far as we know, at least, we first found that zebrafish gene *wfs1b* deficiency robustly suppressed M-cell axon regeneration through regulation in the ER stress pathway, which might shed light on the understanding of new therapeutic strategies not only for WS but also for neurodegeneration (Fig. 8).

In this study, we established a mutant zebrafish line that has a 23-bp deletion in *wfs1b*, facilitating us to investigate how Wfs1b affects M-cell axon regeneration. The whole protein expression level of WFS1, including the protein expression level of Wfs1a and Wfs1b, was decreased. As shown in supplementary, we conducted PCR experiments on the basis of primers spanning the target sequence both at the genome and cDNA levels, and no PCR band was verified in the *wfs1b*^*−/−*^ mutant zebrafish. The *wfs1b* mutant zebrafish exhibited OKR deficiency, by implication, optic dysfunction, which was consistent with previous studies in that the WS patients displayed malfunctions in the visual function [5].

M-cells, the biggest motor neurons that regulate the escape response triggered by threatening events in zebrafish, have a powerful capacity for regeneration after two-photon axotomy [28, 29]. Due to their limited numbers, large size, and ease to view, M-cells became a powerful model for investigating axon degeneration in vivo [32, 37, 38, 55, 56]. Our results revealed that M-cell axon regeneration was intensely inhibited in the *wfs1b*^*−/−*^ mutant zebrafish as well as the function of M-cells, including shorter maximal turn angle and longer duration to maximal turn angle. The length and function of the M-cell axon did not vary from the mutant larvae compared to the wildtype before ablation, that is, inhibition of M-cell was likely due to *wfs1b* deficiency rather than development. Furthermore, the regeneration of the M-cell axon was promoted after complementation of the *wfs1b* gene at a single cell level in the *wfs1b*^*−/−*^ zebrafish larvae, suggesting that *wfs1b* might be an intrinsic factor in regulating axonal regeneration.

ER is an organelle that participates in a wide variety of cellular functions, such as calcium regulation, post-translational modification, and folding of membrane and secretory proteins [26]. WFS1 plays a protective role in regulating ER functions and WS is considered the best prototype for ER diseases since WFS1 is localized in ER [57]. It has been reported that knockdown of WFS1 induced upregulation of ATF6α and its target genes in the rat insulinoma cells [27]. Lack of *wfs1* in mice β-cells induced increased markers of ER stress and abnormal ER morphology [13, 14]. The *wfs1* knockout mice exhibited increased BiP mRNA expression level in arginine vasopressin (AVP) neurons, as BiP has been used as a marker of ER stress [58]. A mass of evidence indicated that a proportion of neurodegenerative diseases are closely related to ER dysfunction, which resulted from exacerbated ER stress [59–61]. Our results showed that the interruption of *wfs1b* gave rise to the upregulation of several UPR-associated genes and impaired the ER structure in zebrafish larvae. Thus, M-cell axon degeneration might be in connection with the *wfs1b* deficiency-mediated ER stress response.

Research has proved that inhibition of ER stress through injecting 4-PBA subconjunctivally could accelerate corneal epithelial wound healing and nerve regeneration [41]. In our study, treating *wfs1b*^*−/−*^ mutant zebrafish larvae with 4-PBA could ameliorate M-cell axon regeneration partly due to alleviated ER stress and 4-PBA itself has no significant effect on M-cell axon regeneration. However, it’s irreversible when *wfs1b* deficiency-induced abnormal ER morphology appeared. Induction of ER stress via administering TM, which causes UPR activation in all species studied [42], could hamper axonal regeneration in *wfs1b*^+*/*+^ zebrafish larvae. It was noteworthy that *wfs1b*^*−/−*^ mutant zebrafish larvae that were treated with TM exhibited an increased ER stress response compared with *wfs1b*^*−/−*^ zebrafish larvae treated with DMSO, suggesting the vulnerability to the disease in the *wfs1b*^*−/−*^ mutant zebrafish, whereas the regenerative length showed no remarkable difference, indicating that a certain degree of ER stress response was enough to induce nerve degeneration.

There are, however, a few limitations. First of all, our study is to emphasize the relationship between the knock-out of *wfs1b*, strictly speaking, knockdown of wfs1 in the zebrafish, and M-cell axon regeneration. Double knock-out of *wfs1a* and *wfs1b* in the zebrafish are encouraged to investigate. Besides, Wfs1b is involved in not only the ER but also the mitochondria [54, 62]. Whether mitochondria are associated with M-cell axon regeneration in the *wfs1b*^*−/−*^ zebrafish model or not is unknown. Finally, we merely utilized pharmacological approaches to investigate the connection of M-cell axon regeneration with ER stress. It remains to be clarified the underlying molecular pathways of how *wfs1b* deficiency-mediated ER stress renders M-cell axon degeneration.

## Supplementary Information

**Additional file 1**: **Table S1**. Plasmid constructs primer sequences.

**Additional file 2**: **Figure S1**. Agarose gel electrophoresis of the wildtype, heterozygosis, and homozygosis. (**a**) Manipulation of Ecil endonuclease among the wildtype, *wfs1b*^*+/-*^ and *wfs1b*^*-/-*^. (**b**) The targeted fragments were amplified by PCR from genomic DNA and then digested with Ecil. Red arrows represented the shorter cleaved PCR bands, blue arrows represent the longer cleaved PCR bands, and green arrows represented the uncleaved PCR bands. **Figure S2**. Examination of mutations in genomic DNA and cDNA levels. (**a**) Schematic of primers design in genomic DNA. (**b**) PCR bands from genomic DNA. About 1000 bp fragment was amplified. (**c**) Schematic of primers design in cDNA. (**d**) PCR bands from cDNA. About 500 bp fragment was amplified. No PCR band was amplified from genomic DNA and cDNA in *wfs1b* mutant zebrafish. **Figure S3**. *wfs1b* mutant zebrafish showed optokinetic response (OKR) deficiency. (**a**) Schematic of the apparatus used to measure the OKR of zebrafish larvae. (**b**) OKR behavior tests of wildtype and *wfs1b* mutant zebrafish larvae at 5 dpf under 0.04 cycle/degree and 0.6 contrast conditions. wildtype, *n*=12; *wfs1b*^*-/-*^, *n*=7. *P* = 0.0033. Assessed by unpaired *t* test. **Figure S4**. Regulation of heterozygote on M-cell axon regeneration. (**a**) M-cell axon regeneration was hindered in *wfs1b*^*+/-*^ mutant zebrafish in vivo. (**b**) the regenerative length of the M-cell axons at 2 dpa. White asterisk: ablation point. Violin plot shows all data points, including minimum, maximum, median, and quartiles. Scale bar, 20 μm. *wfs1b*^*+/+*^, *n*=21; *wfs1b*^*+/-*^, *n*=25. *P* = 0.0004. Assessed by unpaired *t* test. **Figure S5**. qPCR analyses of *atf4b* genes after treatment with TM and 4-PBA. (**a**) Treatment of 4-PBA did not relieve the mRNA expression of *atf4b*. ns, not significant. Assessed by ordinary one-way ANOVA. (**b**) Treatment of TM accelerated the mRNA expression of *atf4b*. Assessed by two-way ANOVA/Tukey’s multiple-comparisons test. **Figure S6**. Electron microscope of zebrafish brain ultrastructure during application of 4-PBA. (**a**–**f**) Representative TEM imaging showed the normal ER morphology in WT (above), swelling and ruptured ER morphology in the *wfs1b* mutant (middle), and 4-PBA – applied (below) zebrafish larvae brain. (a´-f´) were the magnification of the white boxes in (a-f). Scale bar, 500 nm. L, rough endoplasmic reticulum; N, cell nuclei; M, mitochondria. (**g**) Statistical diagram of perimeter and Feret’ diameter among the wildtype +DMSO, *wfs1b*^*-/-*^ +DMSO and *wfs1b*^*-/-*^ +4-PBA groups. Assessed by ordinary one-way ANOVA.

## Data Availability

All data generated or analyzed during this study are included in this published article and its supplementary information files.
