# Supplementary material for: Wolfram syndrome 1b mutation suppresses Mauthner-cell axon regeneration via ER stress signal pathway
Source: Acta Neuropathol Commun. 2022 Dec 17;10:184. doi: 10.1186/s40478-022-01484-8 (PMC9758940; doi:10.1186/s40478-022-01484-8)
Supplement: Supplementary file 1 — Additional file 1: Table S1. Plasmid constructs primer sequences. [file 40478_2022_1484_MOESM1_ESM.docx]

Table S1. Plasmid constructs primer sequences

| Name | Primer sequences (5’->3’) | Usage |
| --- | --- | --- |
| Sg-F | TAATACGACTCACTATAGGGGGCGGAGTCATGGCTGAGTTTTAGAGCTAGAAATAGC | SgRNA  template |
| Sg-R | AGCACCGACTCGGTGCCACT | SgRNA  template |
| anti-*wfs1b*-1-F | TGTGTACCCGTATGGCCACAGC | WISH |
| anti-*wfs1b*-1-R | TAATACGACTCACTATAGGGCACTTCAAACTTGTAGTGGTCAA | WISH |
| anti-*wfs1a*-F | CAGAGAACGCACAGGAGGTGAG | WISH |
| anti-*wfs1a*-R | TAATACGACTCACTATAGGGGGGATAATAGTGCTCAGCCACTG | WISH |
| KO-F | AATTAGCTCAGTCATTTTCAAGAGC | KO test |
| KO-R | CCAGAGGAATAAACCAGAAGAAAT | KO test |
| actin- genome-F | TGATGAAATTGCCGCACTGGT | KO test |
| actin- genome-R | CGTTAACTGGATGTTCAAGTGT | KO test |
| actin- cDNA -F | CCAACTGGGATGATATGGAGA | KO test |
| actin- cDNA -R | CTCTCATTGCCAATGGTGATG | KO test |
| *wfs1b*-genome-F | CCTTCAGCCATGACTCCGCCC | KO test |
| *wfs1b*-genome-R | CAGAGCTTCACCCACCCTGATC | KO test |
| *wfs1b*-cDNA-F | CCTTCAGCCATGACTCCGCCC | KO test |
| *wfs1b*-cDNA -R | TCTTGCGTTCTGGATTGAGTTTCC | KO test |
| *β-actin*-F | CCCTGTTCCAGCCATCCTT | qPCR |
| *β-actin*-R | TTGAAAGTGGTCTCGTGGATACC | qPCR |
| *hspa5*-F | ACGAGAACACAGAAGACGGG | qPCR |
| *hspa5*-R | ATCCCAATCACTGTCCCAAC | qPCR |
| *atf6*-F | CTGTGGTGAAACCTCCACCT | qPCR |
| *atf6*-R | CATGGTGACCACAGGAGATG | qPCR |
| *atf4b*-F | CTTTCTCTCCTCCTGCTTCT | qPCR |
| *atf4b*-R | GAGTCACACGACCCAATCA | qPCR |
| *hsp90b1*-F | GAGGCACCACCATCACGTTG | qPCR |
| *hsp90b1*-R | TCGGTCTTGCTGCTCCATAC | qPCR |
