# Supplementary material for: Wolfram syndrome 1b mutation suppresses Mauthner-cell axon regeneration via ER stress signal pathway
Source: Acta Neuropathol Commun. 2022 Dec 17;10:184. doi: 10.1186/s40478-022-01484-8 (PMC9758940; doi:10.1186/s40478-022-01484-8)
Supplement: Supplementary file 2 — Additional file 2: Figure S1. Agarose gel electrophoresis of the wildtype, heterozygosis, and homozygosis. (a) Manipulation of Ecil endonuclease among the wildtype, wfs1b+/- and wfs1b-/-. (b) The targeted fragments were amplified by PCR from genomic DNA and then digested with Ecil. Red arrows represented the shorter cleaved PCR bands, blue arrows represent the longer cleaved PCR bands, and green arrows represented the uncleaved PCR bands. Figure S2. Examination of mutations in genomic DNA and cDNA levels. (a) Schematic of primers design in genomic DNA. (b) PCR bands from genomic DNA. About 1000 bp fragment was amplified. (c) Schematic of primers design in cDNA. (d) PCR bands from cDNA. About 500 bp fragment was amplified. No PCR band was amplified from genomic DNA and cDNA in wfs1b mutant zebrafish. Figure S3. wfs1b mutant zebrafish showed optokinetic response (OKR) deficiency. (a) Schematic of the apparatus used to measure the OKR of zebrafish larvae. (b) OKR behavior tests of wildtype and wfs1b mutant zebrafish larvae at 5 dpf under 0.04 cycle/degree and 0.6 contrast conditions. wildtype, n=12; wfs1b-/-, n=7. P = 0.0033. Assessed by unpaired t test. Figure S4. Regulation of heterozygote on M-cell axon regeneration. (a) M-cell axon regeneration was hindered in wfs1b+/- mutant zebrafish in vivo. (b) the regenerative length of the M-cell axons at 2 dpa. White asterisk: ablation point. Violin plot shows all data points, including minimum, maximum, median, and quartiles. Scale bar, 20 μm. wfs1b+/+, n=21; wfs1b+/-, n=25. P = 0.0004. Assessed by unpaired t test. Figure S5. qPCR analyses of atf4b genes after treatment with TM and 4-PBA. (a) Treatment of 4-PBA did not relieve the mRNA expression of atf4b. ns, not significant. Assessed by ordinary one-way ANOVA. (b) Treatment of TM accelerated the mRNA expression of atf4b. Assessed by two-way ANOVA/Tukey’s multiple-comparisons test. Figure S6. Electron microscope of zebrafish brain ultrastructure during application of 4-PBA [file 40478_2022_1484_MOESM2_ESM.docx]

Figure S1. Agarose gel electrophoresis of the wildtype, heterozygosis, and homozygosis.


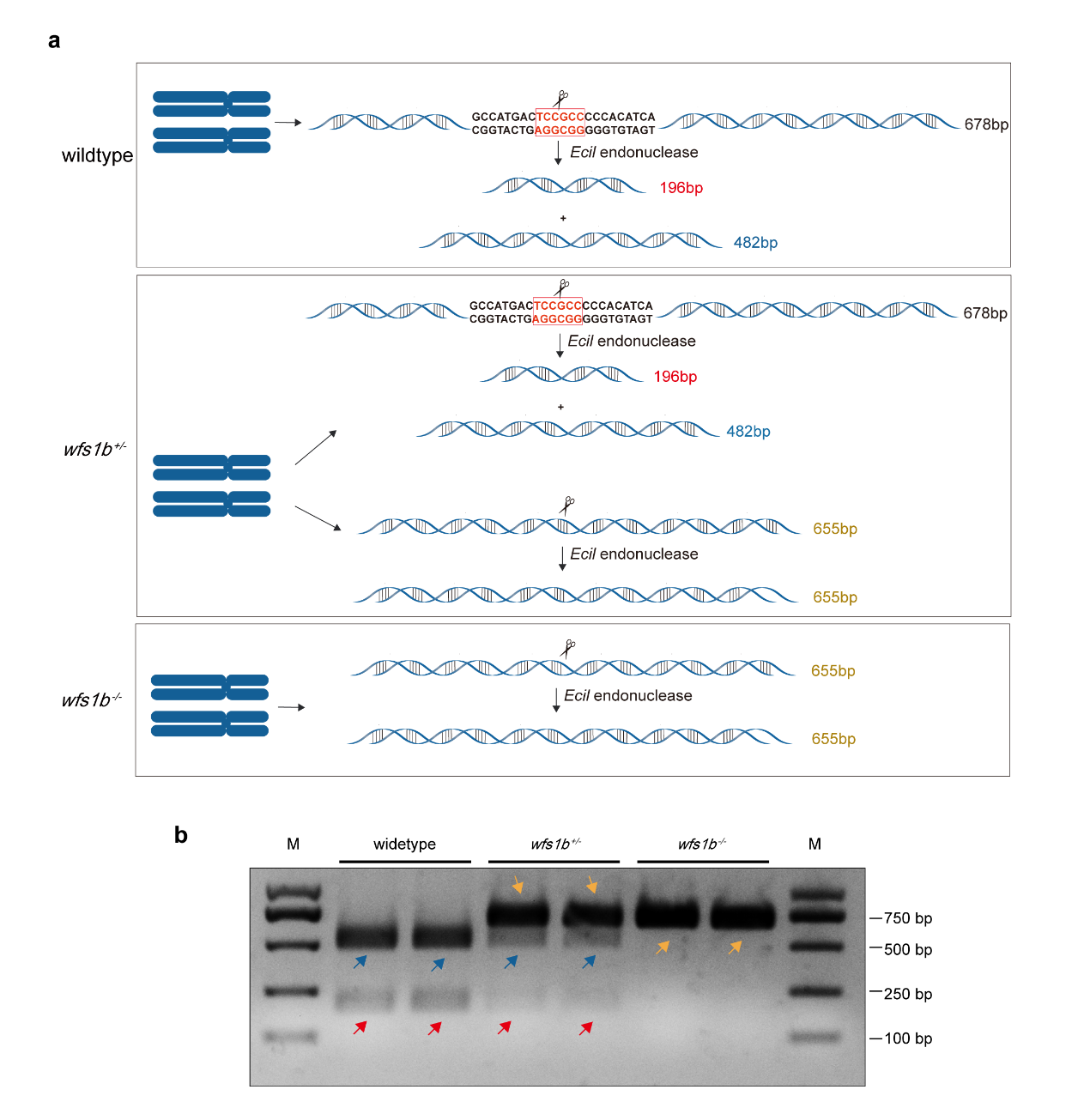


**(a)** Manipulation of Ecil endonuclease among the wildtype, *wfs1b^+/-^* and *wfs1b^-/-^*. **(b)** The targeted fragments were amplified by PCR from genomic DNA and then digested with Ecil. Red arrows represented the shorter cleaved PCR bands, blue arrows represent the longer cleaved PCR bands, and green arrows represented the uncleaved PCR bands.

Figure S2. Examination of mutations in genomic DNA and cDNA levels.


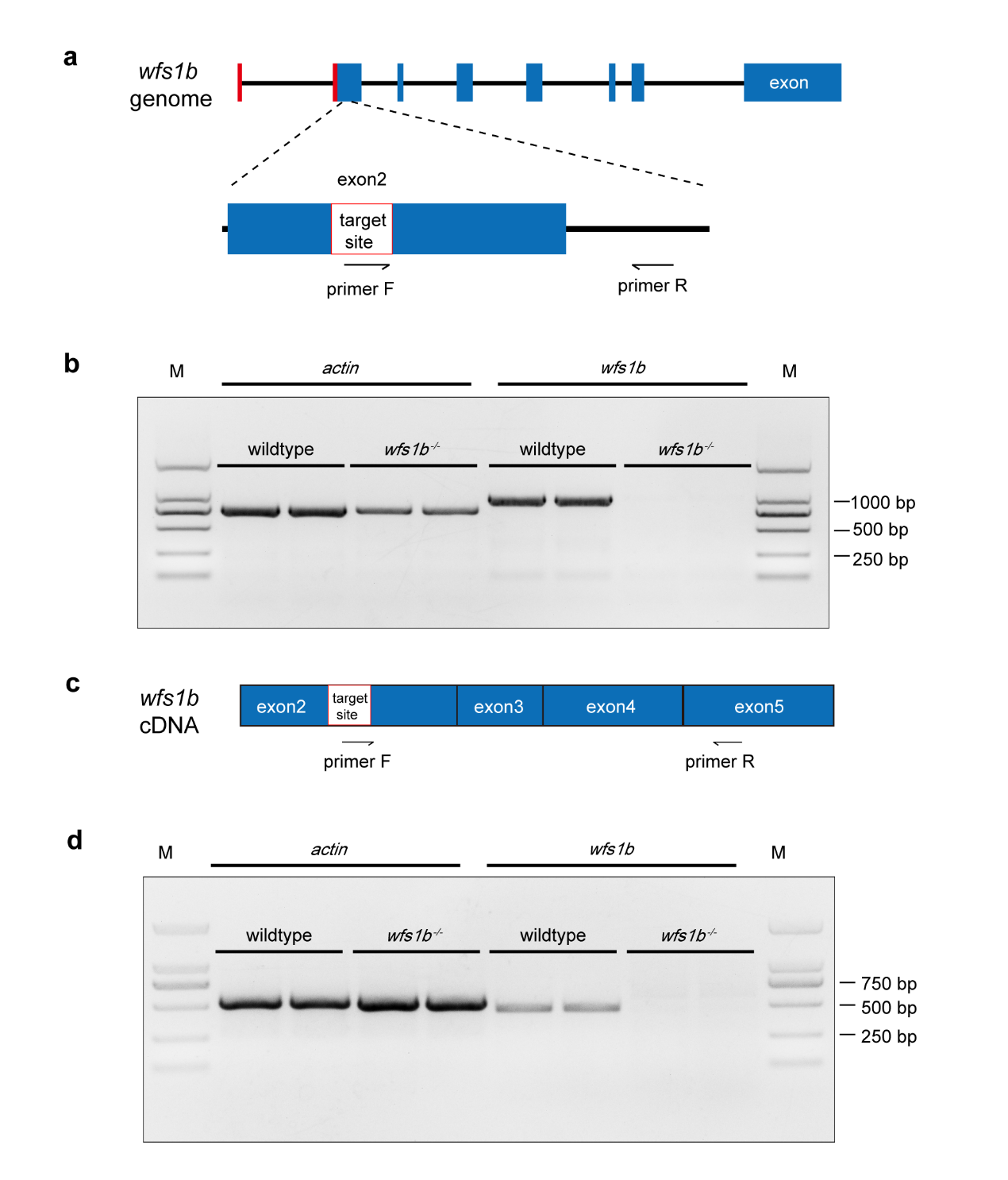


**(a)** Schematic of primers design in genomic DNA. **(b)** PCR bands from genomic DNA. About 1000 bp fragment was amplified. **(c)** Schematic of primers design in cDNA. **(d)** PCR bands from cDNA. About 500 bp fragment was amplified. No PCR band was amplified from genomic DNA and cDNA in *wfs1b* mutant zebrafish.

Figure S3. *wfs1b* mutant zebrafish showed optokinetic response (OKR) deficiency.


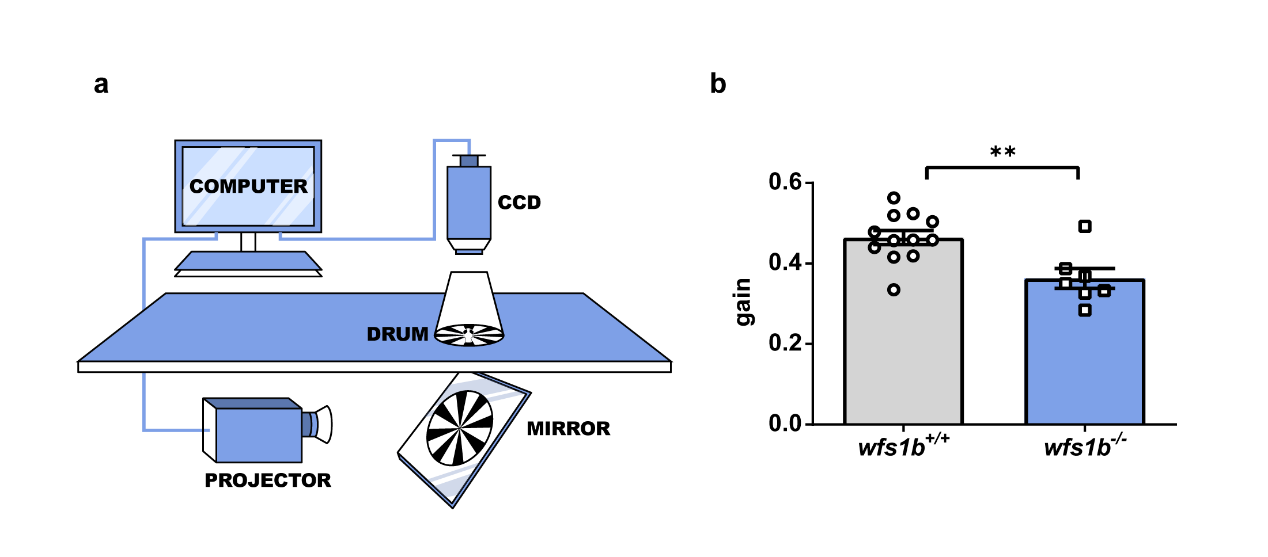


**(a)** Schematic of the apparatus used to measure the OKR of zebrafish larvae. **(b)** OKR behavior tests of wildtype and *wfs1b* mutant zebrafish larvae at 5 dpf under 0.04 cycle/degree and 0.6 contrast conditions. wildtype, *n*=12; *wfs1b^-/-^*, *n*=7. *P* =0.0033. Assessed by unpaired *t* test.

Figure S4. Regulation of heterozygote on M-cell axon regeneration.


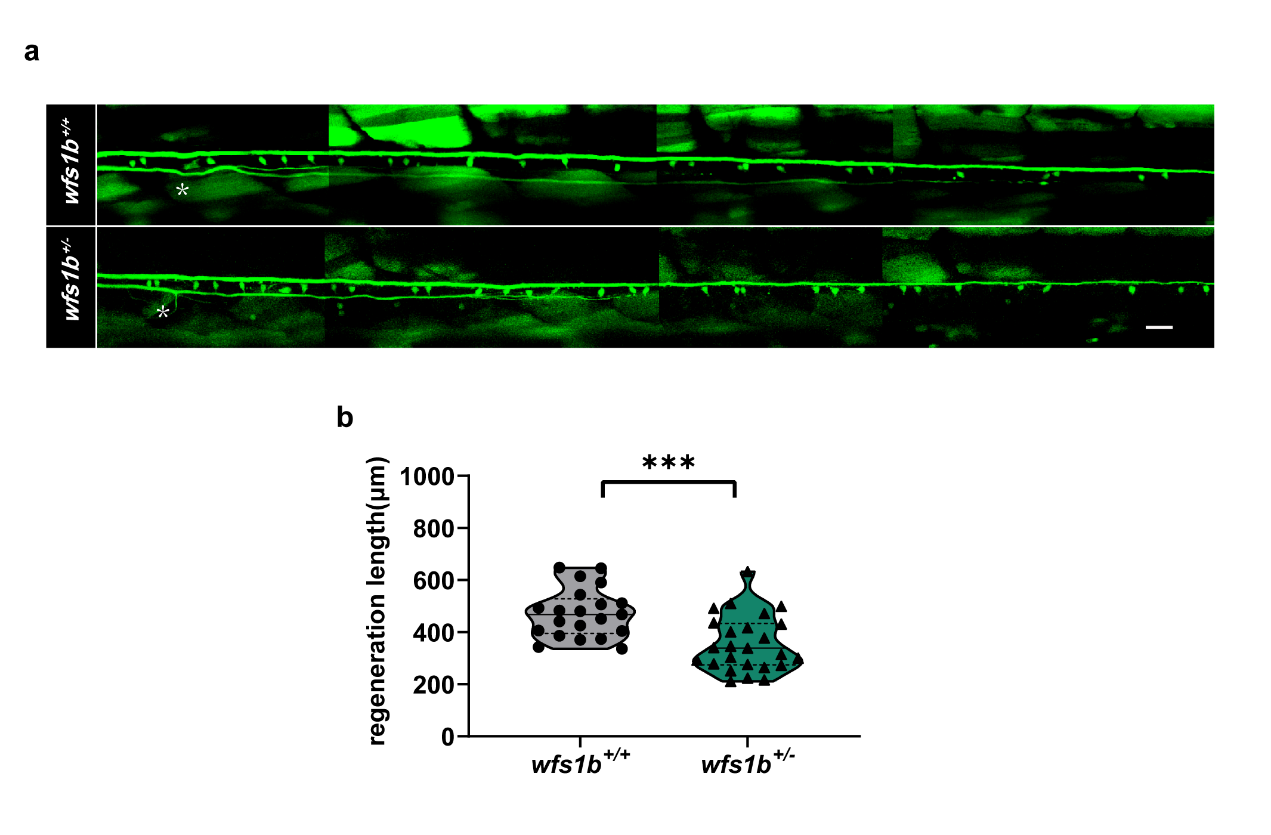


**(a)** M-cell axon regeneration was hindered in *wfs1b^+/-^* mutant zebrafish *in vivo*. **(b)** the regenerative length of the M-cell axons at 2 dpa. White asterisk: ablation point. Violin plot shows all data points, including minimum, maximum, median, and quartiles. Scale bar,20 μm. *wfs1b^+/+^*, *n*=21; *wfs1b^+/-^*, *n*=25. *P* =0.0004. Assessed by unpaired *t* test.

Figure S5. qPCR analyses of *atf4b* genes after treatment with TM and 4-PBA.


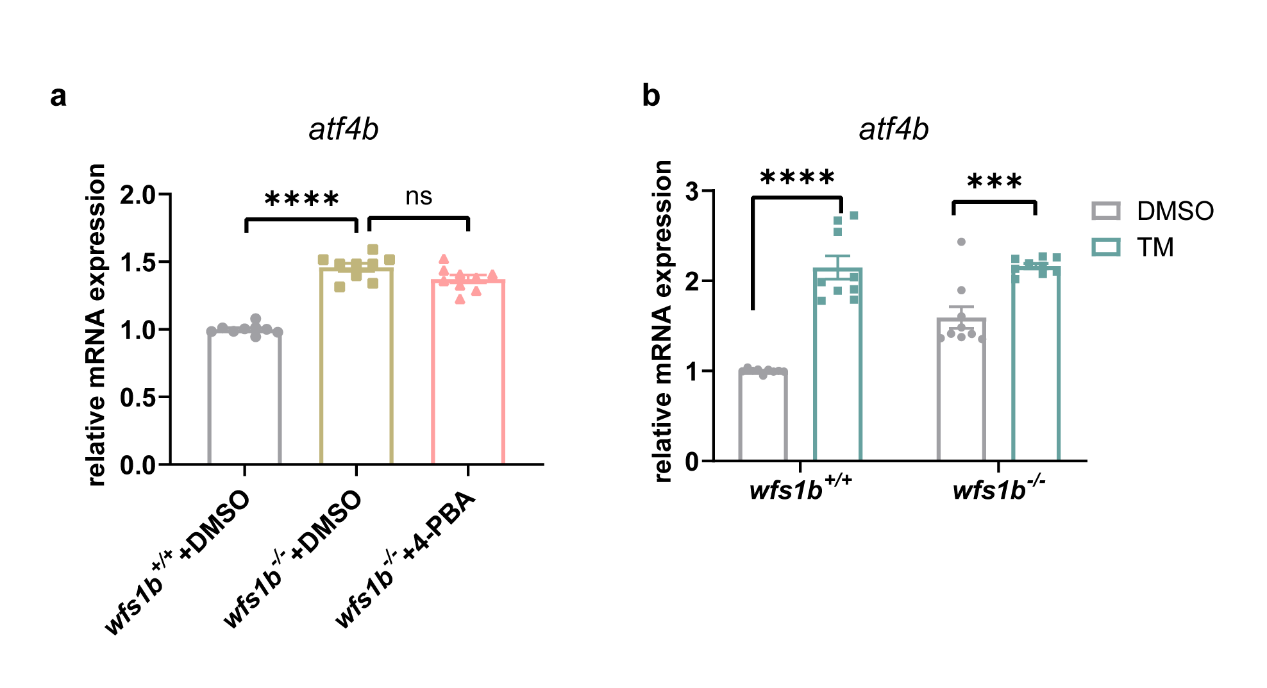


**(a)** Treatment of 4-PBA did not relieve the mRNA expression of *atf4b*. ns, not significant. Assessed by ordinary one-way ANOVA. **(b)** Treatment of TM accelerated the mRNA expression of *atf4b*. Assessed by two-way ANOVA/Tukey’s multiple-comparisons test.

Figure S6. Electron microscope of zebrafish brain ultrastructure during application of 4-PBA.


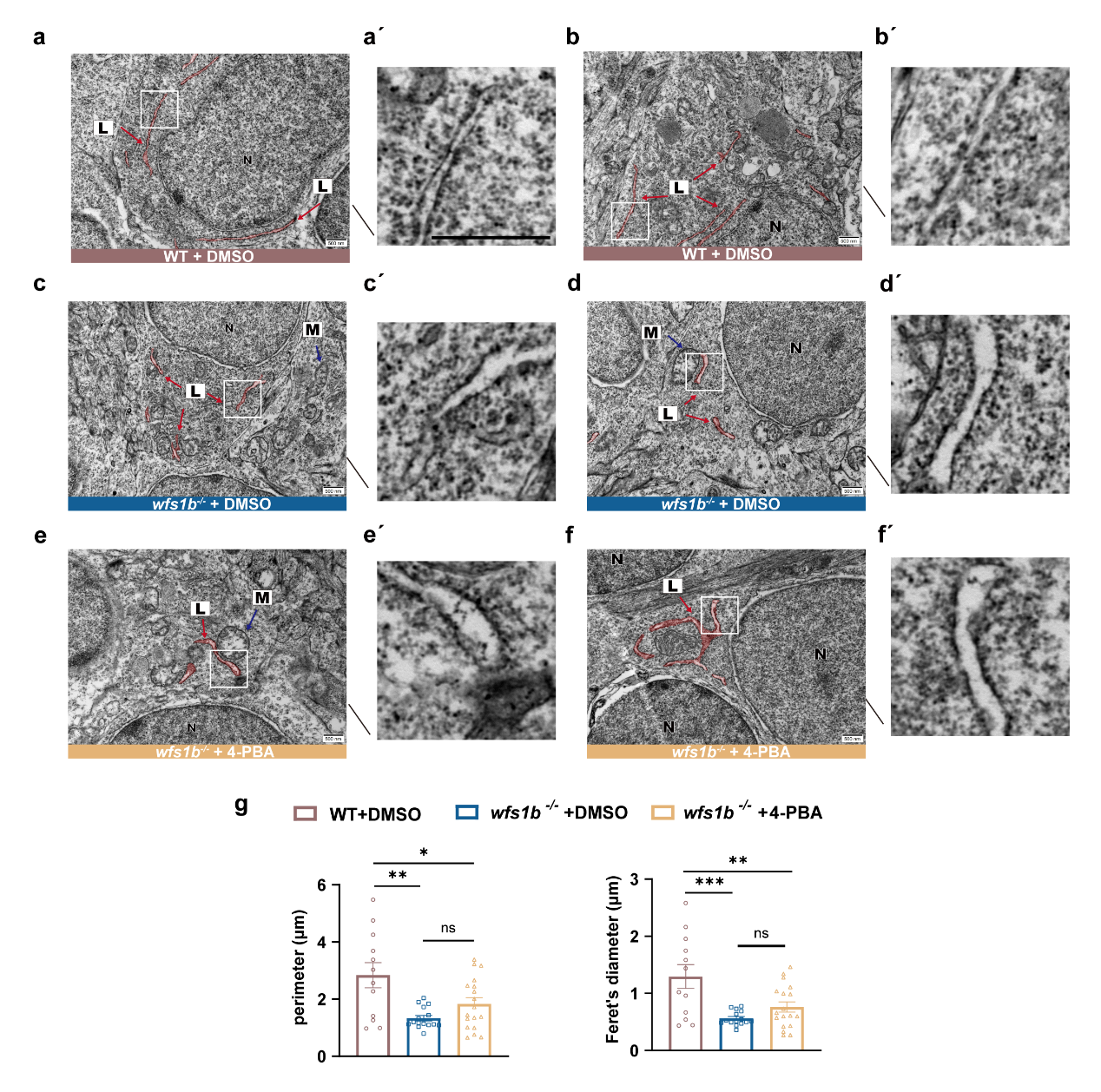


**(a-f)** Representative TEM imaging showed the normal ER morphology in WT (above), swelling and ruptured ER morphology in the *wfs1b* mutant (middle), and 4-PBA – applied (below) zebrafish larvae brain. (a´-f´) were the magnification of the white boxes in (a-f). Scale bar, 500 nm. L, rough endoplasmic reticulum; N, cell nuclei; M, mitochondria. **(g)** Statistical diagram of perimeter and Feret’ diameter among the wildtype +DMSO, *wfs1b^-/-^* +DMSO and *wfs1b^-/-^* +4-PBA groups. Assessed by ordinary one-way ANOVA.
